# Thrombin based gelatin matrix and fibrin sealant mediated clot formation in the presence of clopidogrel

**DOI:** 10.1186/1477-9560-12-10

**Published:** 2014-05-07

**Authors:** Joseph F Dwyer, Jill A McCoy, Ziping Yang, Michael Husser, Heinz Redl, Mary Ann Murphy, Martin Wolfsegger, James P DiOrio, Andreas Goppelt, Shane Donovan

**Affiliations:** 1 Baxter Healthcare Corporation, Deerfield, IL, USA; 2 Ludwig Boltzmann Institute for Experimental and Clinical Traumatology, AUVA Research Center, Austrian Cluster for Tissue Regeneration, Vienna, Austria; 3 Baxter Innovations GmbH, Wagramerstrasse 17-19, 1220 Wien, Austria

**Keywords:** Floseal, Tisseel, Clopidogrel, Thrombelastography, Thrombin, Hemostasis

## Abstract

**Background:**

Platelet inhibitors are commonly used to reduce the risk of atherothrombotic events. The aim of this study was to determine the impact of platelet inhibitors, specifically clopidogrel and aspirin, on clot kinetics, strength, and/or structure during the use of thrombin based gelatin matrices and fibrin sealants.

**Methods:**

Blood was collected and heparinized from donors on clopidogrel (and aspirin) and age matched control donors. Blood component analysis, whole blood platelet aggregometry, and activated clotting time (ACT) were used to monitor compliance to therapy and identify any differences between donor groups. Clot kinetics and strength were analyzed using thrombelastography (TEG). Field Emission Scanning Electron Microscopy (FESEM) was used to analyze clot structure.

**Results:**

Blood component profiles were similar for both donor groups. Aggregometry indicated that aggregation response to adenosine diphosphate (ADP) for clopidogrel donors was 12% of that for the controls (p = 0.0021), an expected result of clopidogrel induced platelet inhibition. However, blood from both donor groups had an elevated thrombin induced aggregation response. Heparinization of donor blood resulted in similarly elevated ACTs for both donor groups. TEG results indicated similar clot kinetics and strength between clopidogrel and control donor groups for blood alone and when clotting was induced using thrombin based gelatin matrices and fibrin sealants. FESEM images supported TEG findings in that similar morphologies were observed in *ex vivo* formed clots from both donor groups when thrombin based gelatin matrices and fibrin sealants were used.

**Conclusion:**

These results suggest that platelet inhibitors do not negatively impact clot kinetics, strength, and structure when clotting is initiated with thrombin based gelatin matrices and fibrin sealants.

## Background

Platelets contribute to physiological hemostasis by aggregating at the site of a lesion, providing surfaces for hemostatic reactions, and supplying regulatory hemostatic factors. They also contribute to atherosclerotic disease by stimulating and stabilizing thrombi. Following myocardial infarction or stroke, the American Heart Association and American College of Cardiology guidelines recommend that patients take platelet inhibitors to reduce the risk of further atherothrombotic events [1,2]. Clopidogrel and aspirin (acetylsalicylic acid) are commonly used platelet inhibitors. Both drugs reduce mortality in patients with chronic atherosclerotic disease and are also effective drugs for the acute treatment of infarcts [3].

Platelet aggregation is disrupted by these inhibitors via two distinct pathways. Clopidogrel acts as an inhibitor of adenosine diphosphate (ADP) induced platelet aggregation by covalently binding to its receptor, P2Y_12_. ADP mediated activation of the GPIIb/IIIa complex is therefore abated [4-6]. Aspirin irreversibly inhibits cyclooxygenase enzymes, COX1 and COX2 [7]. This prevents the conversion of arachidonic acid to thromboxane A_2_ and subsequent thromboxane stimulated platelet aggregation [8,9]. Clopidogrel and aspirin can be used individually or in combination for dual anti-platelet therapy.

Several clinical studies have linked the use of platelet activation inhibitors to increased bleeding during surgery resulting in excess blood loss, increased transfusion rates, and reoperation [10-13]. These findings led the authors of these studies to recommend that surgery be delayed if possible to allow patients to withdraw from anti-platelet therapy [10,12]. Clopidogrel and aspirin both irreversibly alter platelet function. As a result, platelet aggregation and bleeding time generally do not return to normal levels until 5 days after discontinuing usage when sufficient quantities of new unaffected platelets have been produced [14]. Recommendations for and optimal time interval between cessation of anti-platelet medications and surgery vary from 3–10 days [15-18].

Other studies have suggested that the risk of further atherothrombotic events outweighs the risk of increased bleeding during surgery and recommend that in certain situations patients remain on either aspirin or dual anti-platelet therapy throughout the perioperative period [19,20]. For example, patients who are at an intermediate or high risk for cerebro- or cardiovascular events are recommended to remain on both clopidogrel and aspirin during many types of surgeries such as orthopedic surgery, reconstructive surgery, and endoscopy [19]. Consequently, many patients are undergoing surgical procedures with significant platelet inhibition as a result of a reluctance or inability (i.e. non-elective/emergency procedures) to withhold anti-platelet therapy prior to surgery. In such cases, surgeons face concerns that adjunctive thrombin based gelatin matrix and fibrin sealant hemostatic agents may not work effectively for patients on anti-platelet therapy.

Gelatin matrices and fibrin sealants that contain a thrombin component have been used for many years as effective adjunctive hemostats in surgery [21,22]. Both exogenous hemostats stop bleeding by inducing physiological hemostasis in a number of distinct ways. They curtail bleeding on tissue surfaces by generating fibrin mediated blood clots. As thrombin based gelatin matrices rely on incoming blood to supply the substrates for hemostatic clots, one potential clinical concern is that these hemostats may not work effectively for patients taking platelet aggregation inhibitors such as clopidogrel and aspirin. They also provide a high dose of thrombin, novel topologies, and substrates for blood and tissue derived factors to regulate hemostasis, and, in the case of thrombin based gelatin matrix hemostats, the gelatin particles provide a tamponade effect at the wound site, as well as contact activation, which leads to platelet activation.

The purpose of this study was to evaluate clots formed when thrombin based gelatin matrices and fibrin sealants were mixed with heparinized blood from patients who were taking clopidogrel to determine whether there was a negative impact of this platelet activation inhibitor on clot kinetics, strength, and/or structure. Assays employing whole blood were selected where appropriate for sample testing to more closely reflect the surgical conditions during application of the hemostats. Clot kinetics and strength were analyzed using thrombelastography (TEG) and clot structure was analyzed using Field Emission Scanning Electron Microscopy (FESEM). TEG was deemed an appropriate assay since it uses whole human blood, and it has been used to monitor patient hemostasis profiles during cardiac and general surgery [23,24].

## Methods

### Donor criteria and collection of whole blood

Donor samples were collected from individuals in accordance with Baxter Healthcare’s Internal Review Board. Blood was collected from three donors on clopidogrel and aspirin and one donor on only clopidogrel. Blood was also taken from four age matched control donors who were not taking clopidogrel or aspirin (i.e. control). The small number of donors is a consequence of the limited available donor pool and selection criteria (Table 1). These criteria were used isolate the impact of clopidogrel on hemostat performance from other drugs or medical conditions that interfere with hemostasis. The first 2–3 ml of blood collected was discarded from each donor. Thereafter, 25 ml of blood was collected in sodium citrate (3.2%). An additional 5 ml aliquot was collected in EDTA (1.5 mg/ml). The blood collected in EDTA was used for analysis of the blood cells, platelets, hematocrit, and hemoglobin. Other analyses were performed using citrated blood. All researchers performing the assays were blinded to donor status throughout the course of the study.

**Table 1:** Inclusion and exclusion criteria for donors

**Inclusion criteria for clopidogrel donors**	**Inclusion criteria for age matched control donors**	**Exclusion criteria**
Clopidogrel or clopidogrel and aspirin use for at least 14 days prior to blood draw	No clopidogrel use for at least 14 days prior to blood draw	History of diabetes, renal failure, acute or chronic liver disease, including acute or chronic hepatitis or cirrhosis
If on aspirin, daily aspirin dosage of at least 75 mg/day	No aspirin use for at least 14 days prior to blood draw	Positive for HIV, hepatitis B, or hepatitis C
Males and Females	Males and Females	Use of Selective Serotonin Re-uptake Inhibitors (SSRIs), Seritonin-Norepinepherin Re-uptake Inhibitors (SNRIs), Omeprazole (which interferes with clopidogrel metabolism) [25], or oral anticoagulants (Coumadin, Warfarin, etc.)

### Blood component analyses

To investigate possible non-pharmaceutical related differences between the donor groups, a series of blood analyses were performed. Red and white blood cells, platelets, hemoglobin, and hematocrit were quantified for each donor sample with an ADVIA 2120 Hematology System (Siemens Corporation; Malvern, PA) using an aliquot of whole blood freshly collected in EDTA. The remaining blood analyses were performed using plasma prepared by centrifugation of citrated whole blood. The plasma was stored at −80°C. Fibrinogen concentration was measured using a STA Compact Hemostasis System (Diagnostica Stago; Parsippany, NJ), Factor VIII activity was measured using a Behring Coagulation System (Siemens Corporation; Malvern, PA), and Factor XIII levels were measured by ELISA (AssayPro; St. Charles, MO).

### Whole blood platelet aggregometry

Platelet aggregation induced by either ADP (10 μM final concentration, Chrono-Log Corporation; Havertown, PA.) or Thrombin (1 IU/ml final concentration, Baxter Healthcare Corporation; Deerfield, IL) was assessed in citrated whole blood on both clopidogrel and control donors. A Model 560VS Dual Channel Whole Blood Lumi-Aggregometer (Chrono-log; Havertown, PA) using an impedance technique was used for testing each sample in duplicate per agonist, with the duplicates being averaged to obtain a final result.

### Blood heparinization and activated clotting time (ACT)

Heparin (APP Pharmaceuticals, LLC; Schaumburg, IL) was added to each citrated whole blood sample to achieve a final concentration of 5 U/ml prior to TEG and imaging in order to assess the blood under clinically relevant heparin levels [26]. An Activated Clotting Time (ACT) measurement was taken of each blood sample before and after adding heparin using a Hemochron Response Whole Blood Coagulation System (International Technidyne Corporation; Edison, NJ). Because citrated blood was used for these measurements, calcium chloride was added to each test sample immediately prior to ACT analysis to return calcium ion concentration to a sufficient level to overcome the chelating effect of the sodium citrate.

### Thrombelastography (TEG)

#### TEG background

Clot kinetics and strength were measured using a Thrombelastograph 5000 (Haemoscope Corporation; Niles, IL). TEG measurements were taken from citrated whole blood, heparinized blood, heparinized blood that was mixed with a thrombin based gelatin matrix (Floseal VH S/D: Baxter Healthcare Corporation; Deerfield, IL), and heparinized blood mixed with fibrin sealant (Tisseel VH S/D: Baxter Healthcare Corporation; Deerfield, IL). A sufficient level of calcium ion to overcome the chelating effect of sodium citrate was supplied by the thrombin component of each hemostat which was reconstituted in calcium chloride so no further calcium supplementation was needed. The clotting process as monitored by the TEG is reported as a set of coagulation parameters (Table 2).

**Table 2:** Evaluated TEG coagulation parameters

**Coagulation parameter**	**Description**
R-time (R)	The time from the start of a sample run until the first significant levels of detectable clot formation in minutes (min).
K-time (K)	The time from the measurement of R until a fixed level of clot firmness is reached (Amplitude = 20 mm) in minutes (min).
Angle (α)	An indicator of the kinetics of clot formation and fibrinogen level quantified in degrees (º).
Maximum Amplitude (MA)	A measurement of maximum strength or stiffness of the developed clot in millimeters (mm) and an indicator of platelet function.
Shear Elastic Modulus Strength (G)	A measurement of clot strength calculated as G = (5000A/(100-A))/1000 and reported as dynes per square centimeter (d/sc).
Time to MA (TMA)	The time until the maximal clot strength (MA) is reached in minutes (min).

#### TEG analysis with citrated and heparinized blood alone

The cup & pin were loaded onto the TEG. Calcium chloride solution (20 μl) was added to the cup. Citrated or heparinized blood (340 μl) was then added to the cup, and the TEG analysis was immediately initiated. Donor samples were analyzed in triplicate.

#### Preparation of the thrombin based gelatin matrix for TEG analysis

Floseal VH S/D is a bovine-derived gelatin matrix that is prepared with human thrombin and indicated for hemostasis when control of bleeding by ligature or conventional procedures is ineffective or impractical [27]. Three lots of thrombin based gelatin matrix were prepared per IFU (instructions for use) but on a reduced scale. The gelatin (0.100 g or ~1/8 of a 5 ml kit syringe) was weighed out on weigh paper and then back-loaded into a 1 ml tuberculin syringe. To remove air, the granules were compressed towards the luer end of the syringe. In a second tuberculin syringe, 0.5 ml of 500 IU/ml thrombin solution was withdrawn (prepared by adding 5 ml CaCl_2_ to the thrombin vial) and a syringe connector was attached (luer end facing up). The two syringes were connected (one with gelatin and one with thrombin solution) and ‘swooshed’ at least 20 times to mix the gelatin and thrombin solution adequately.

#### TEG analysis with thrombin based gelatin matrix

The cup & pin were loaded onto the TEG. The cup was subsequently removed from the TEG. The TEG cup was placed on the scale and the weight was zeroed. The first 100 μl of mixed thrombin based gelatin matrix was discarded from the syringe. The tip of the syringe was cleaned with a Kimwipe to ensure that no excess thrombin based gelatin matrix remained. Using the tuberculin syringe, 150 μl of the mixed thrombin based gelatin matrix was placed at the bottom of the TEG cup. The weight of the added thrombin based gelatin matrix was recorded. If the weight was 0.1160 g to 0.1260 g, the sample was used. If not, the weight was adjusted so that the weight of sample in the cup was in this range. The cup was impacted against the lab bench several times to concentrate the thrombin based gelatin matrix evenly on the bottom of the cup. Heparinized blood (210 μl) was added to the cup and mixed quickly with the thrombin based gelatin matrix using a pipette tip. The cup was then loaded onto the TEG with analysis initiated immediately. Donor samples were analyzed in triplicate with each of the three lots of thrombin based gelatin matrix.

#### Preparation of the fibrin sealant for TEG analysis

Tisseel VH S/D is a human-derived fibrin sealant indicated for hemostasis in surgeries involving cardiopulmonary bypass and treatment of splenic injuries due to blunt or penetrating trauma when control of bleeding by conventional techniques is ineffective or impractical [28]. Three lots of fibrin sealant were prepared per IFU. The fibrinogen and aprotinin components were warmed to 37°C, and the 40 mM CaCl_2_ was brought to room temperature. The fibrinogen was reconstituted in aprotinin (F.C. ~100 mg/ml). The re-suspended fibrinogen was placed in a Fibrinotherm (Baxter Healthcare Corporation; Deerfield, IL) until dissolved and stored at 37°C until used. The thrombin was reconstituted in 40 mM CaCl_2_ (F.C. ~500 IU/ml) and stored at room temperature until used.

#### TEG analysis with fibrin sealant

The cup & pin were loaded onto the TEG. The cup was subsequently removed from the TEG. Heparinized blood (210 μl) was added to the cup. Fibrinogen solution (75 μl, ~7.5 mg) and thrombin solution (75 μl, ~37.5 IU) were added to the TEG cup simultaneously in conjunction with the blood volume above to total 360 μl. The sample was mixed quickly using a pipette tip and the cup loaded into the TEG. TEG analysis was immediately initiated. Donor samples were analyzed in triplicate with each of the three lots of fibrin sealant.

### Field emission scanning electron microscopy (FESEM)

#### Preparation of thrombin based gelatin matrix clots

The thrombin based gelatin matrix was prepared per IFU as indicated above and 150 μl was dispensed into a well of a 24 well plate. Heparinized blood (210 μl) was added to the well and the blood and hemostat were quickly mixed. The clot was allowed to set for 30 minutes at 37°C before the addition of 2 ml/well of phosphate buffered saline (PBS). Clots were subsequently detached from the bottom of each well. After another 30 minutes at 37°C, the clot was rinsed with 1 ml PBS and fixed overnight at 4°C in 2 ml of 2% glutaraldehyde (Electron Microscopy Sciences; Hatfield, PA) in 0.1 M HEPES buffer.

#### Preparation of fibrin sealant clots

The fibrinogen and thrombin components of the fibrin sealant were prepared per IFU as indicated above. Heparinized blood (210 μl) was added to the well of a 24 well plate followed by 75 μl of fibrinogen solution and 75 μl of thrombin solution. The blood and hemostat were quickly mixed and the clot was allowed to set for 30 minutes at 37°C. Two milliliters of PBS were added to the well and the clot was detached from the bottom of the well. After another 30 minutes at 37°C, the clot was rinsed with 1 ml PBS and fixed overnight at 4°C in 2 ml of 2% glutaraldehyde in 0.1 M HEPES buffer.

#### Preparation of clots for FESEM

After clot fixation, clots were rinsed with 0.1 M HEPES buffer and dehydrated in a graded ethanol (EtOH) series to 100% EtOH. The specimens were placed into 2:1, 1:1, and 1:2 solutions of 100% EtOH to hexamethyldisilazane (HMDS, Electron Microscopy Sciences; Hatfield, PA), rinsed three times in 100% HMDS followed by a quick change of 100% HMDS, and allowed to air dry. The specimens were then mounted onto FESEM stubs affixed with double-stick conductive carbon tape and sputter coated with palladium in a Desk IV Sputter/Etch Unit (Denton Vacuum; Moorestown, NJ). The samples were examined in a JSM-6300 F FESEM (JEOL; Tokyo, Japan) and representative areas were recorded as digital images.

### Statistical analyses

Comparison of clopidogrel versus control results was considered to be exploratory; therefore, analysis focused on estimation of group effects using confidence intervals (CIs) rather than on hypothesis testing. All analyses were performed with R version 2.15.3 (R Core Team; Vienna, Austria) [29].

#### Blood analysis

Differences in blood parameters between clopidrogel and control donors were assessed using ratios of averages, corresponding two-sided 95% CIs, and two-sided p-values obtained by R function “t.test.ratio (option var.equal = FALSE)” of R package “mratios” [30].

#### Thrombelastography

TEG data were analyzed using a linear mixed effects model taking the replicate measurements per donor adequately into account. The model consisted of the fixed effect group (clopidogrel and control) and donor as random effects, and was fitted using R function “lme” of R package “nlme” [31]. Differences in TEG data between clopidogrel and control donors were assessed using ratios of averages, corresponding two-sided 95% CIs and two-sided p-values where the two-sided 95% CI for the ratio of averages were obtained using Fieller’s theorem [32].

## Results

### Donor criteria

Four clopidogrel donors and four control donors that met the set criteria were used for this study. The four clopidogrel donors were taking clopidogrel daily and three of the four donors were also taking daily aspirin. The control donors had not taken clopidogrel or aspirin for at least 14 days prior to blood collection. The median age of clopidogrel and control donors was 55 (range: 47 to 62) and 55 (range: 50 to 58) years, respectively.

### Blood component analyses

**Figure 1:**
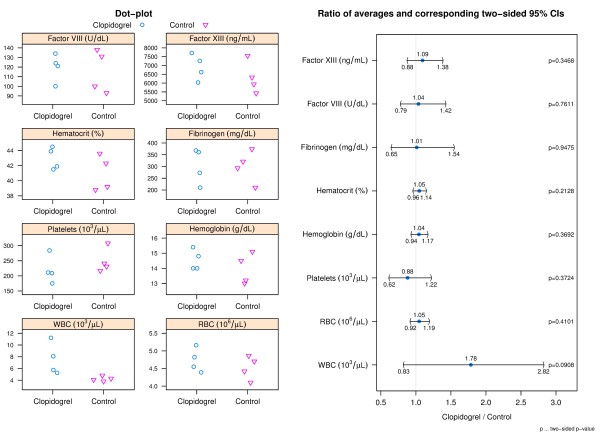
**Blood component analyses results.** The left panel shows dot-plots of all blood parameters and group. The right panel shows a CI plot where blood parameters are indicated on the y-axis and the ratios of averages (clopidogrel relative to control) and corresponding two-sided 95% CIs on the x-axis.

The results of the blood component analyses were similar between donor groups (Figure 1). The largest difference between donor groups was observed in WBC with a 1.78 (95% CI: 0.83 to 2.82) times higher average WBC count for clopidogrel donors than for control donors; however, this difference was not statistically significant at the 5% level.

### Whole blood platelet aggregometry

**Figure 2:**
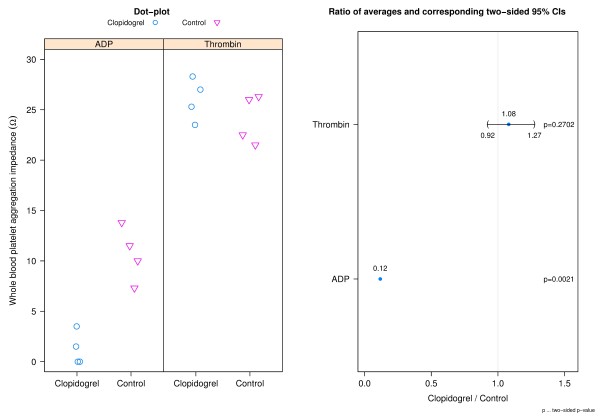
**Whole blood aggregation results after activation with ADP or thrombin.** The left panel shows dot-plots of whole blood platelet aggregation impedance per group for aggregation induced with ADP and thrombin. The right panel shows a CI plot where the type of induction is indicated on the y-axis and the ratios of averages (clopidogrel relative to control) and corresponding two-sided 95% CIs are presented on the x-axis. No bounded two-sided 95% CI for the ratio regarding ADP is available as the numerator of the ratio (i.e. mean of clopidrogel) is not statistically different from zero.

Average aggregation response to adenosine diphosphate (ADP) for clopidogrel donors was 12% of that for the controls (p = 0.0021) (Figure 2). However, when thrombin was used as the agonist on the same blood obtained from the same donors, the platelet aggregation response was normalized with a 1.08 (95% CI: 0.92 to 1.27) times larger average response for clopidogrel donors.

### Activated clotting time (ACT)

**Figure 3:**
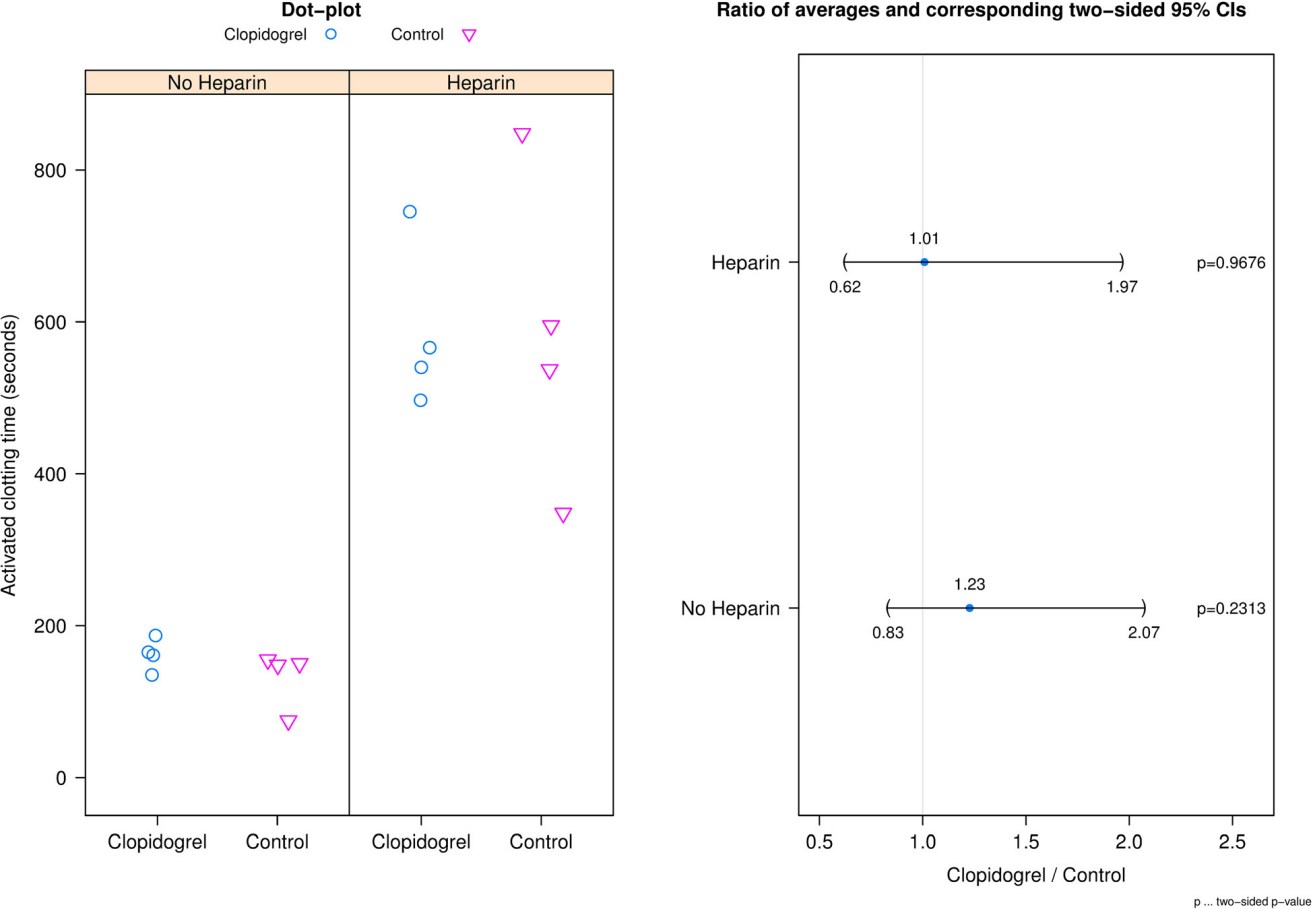
**ACT results pre and post-heparinization.** The left panel shows dot-plots of activated clotting time per group and pre and post-heparinization. The right panel shows a CI plot for pre- and post-heparinization (y-axis) where the ratios of averages (clopidogrel relative to control) and corresponding two-sided 95% CIs are presented on the x-axis.

The addition of heparin to the each donor’s blood resulted in an expected increase in ACT (Figure 3). On average, clopidogrel donors had a 1.23 (95% CI: 0.83 to 2.07) and 1.01 (95% CI: 0.62 to 1.97) times longer ACT before and after addition of heparin, respectively.

### Thrombelastography (TEG)

#### Citrated and heparinized blood alone

**Figure 4:**
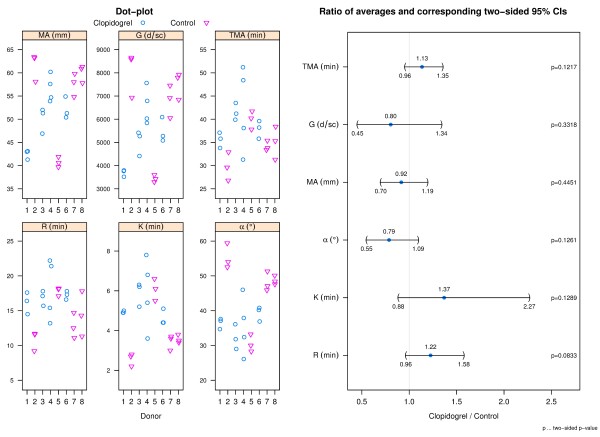
**TEG data: citrated blood.** The left panel shows dot-plots of all TEG parameters per donor and group. The right panel shows a CI plot where TEG parameters are indicated on the y-axis and the ratio of averages (clopidogrel relative to control) and corresponding two-sided 95% CIs on the x-axis.

TEG data for citrated blood (Figure 4) indicates that across all TEG parameters there is a trend which suggests that clopidogrel may impede hemostasis; however differences between donor groups did not reveal statistical significance at the 5% level for any of the parameters. Heparinized blood alone samples did not clot for any donor and were stopped after 45 minutes in the TEG (data not shown).

#### Thrombin based gelatin matrix and fibrin sealant

**Figure 5:**
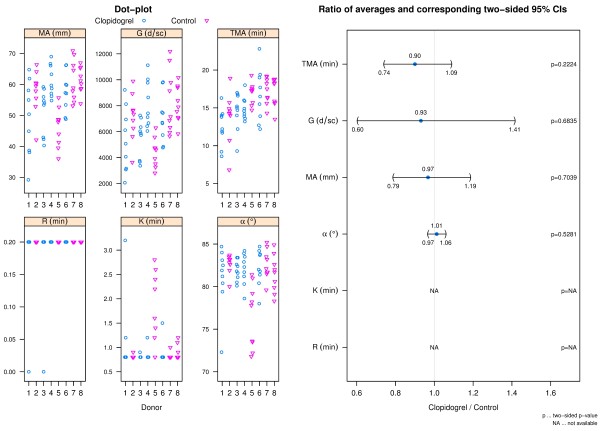
**TEG data: thrombin based gelatin matrix.** The left panel shows dot-plots of all TEG parameters per donor and group. The right panel shows a CI plot where TEG parameters are indicated on the y-axis and the ratio of averages (clopidogrel relative to control) and corresponding two-sided 95% CIs on the x-axis. Due to the rapid rate of clot formation when using the thrombin based gelatin matrix, the TEG is not sensitive enough to generate values for R and K other than the lowest possible data value for most sample replicates preventing comparison these parameters between donor groups.

**Figure 6:**
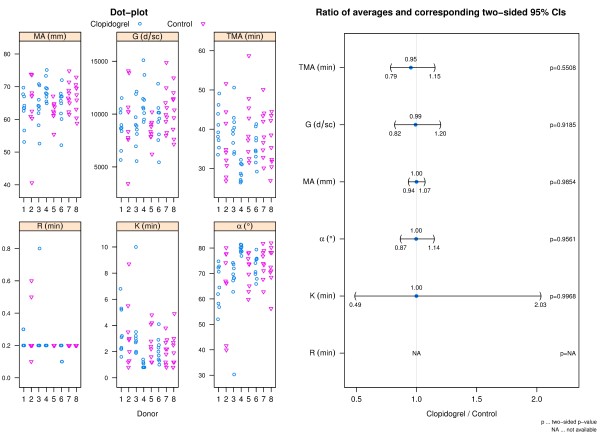
**TEG data: fibrin sealant.** The left panel shows dot-plots of all TEG parameters per donor and group. The right panel shows a CI plot where TEG parameters are indicated on the y-axis and the ratio of averages (clopidogrel relative to control) and corresponding two-sided 95% CIs on the x-axis. Due to the rapid rate of clot formation when using the fibrin sealant, the TEG is not sensitive enough to generate values for R other than the lowest possible data value for most sample replicates preventing comparison this parameters between donor groups.

TEG parameter results for the thrombin based gelatin matrix (Figure 5) and fibrin sealant (Figure 6) mixed with donor blood were similar between donor groups based on the ratios of averages.

### FESEM of thrombin based gelatin matrix and fibrin sealant clots

**Figure 7:**
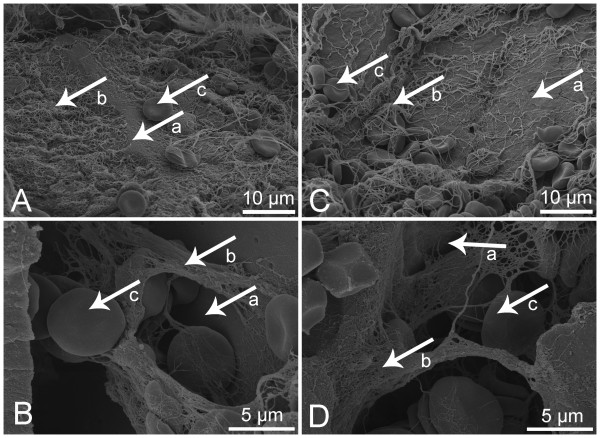
**Representative images of thrombin based gelatin matrix clots acquired with FESEM.** Control **(A,B)**, clopidogrel **(C,D)**; 10 μm scale bars **(A,C)**, 5 μm scale bars **(B,D)**; Arrows: Gelatin (a), Fibrin (b), Red Blood Cells (c). No qualitative differences were observed in the fibrin structure (porosity, fiber thickness, and branching) or the cellular accumulation around the gelatin granules including large numbers of trapped RBCs between clopidogrel and control donor groups.

**Figure 8:**
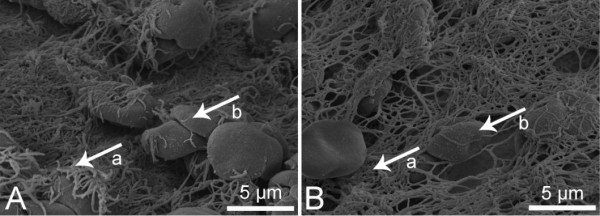
**Representative images of fibrin sealant clots acquired with FESEM.** Control **(A)**, clopidogrel **(B)**; 5 μm scale bars; Arrows: Fibrin (a), Red Blood Cells (b). As seen in the images of the thrombin based gelatin matrix clots, no qualitative differences were observed between clopidogrel and control donor groups. The density of the fibrin structure seen compared to the gelatin based hemostat clots may be due to the additional fibrinogen supplied by the fibrin sealant.

Images of thrombin based gelatin matrix clots (Figure 7) from both donor groups appeared morphologically similar. The fibrin structure had similar fiber thickness, branching, and porosity. Clots generated with the fibrin sealant (Figure 8) also had similar morphology for both donor groups.

## Discussion

In this study, clopidogrel had no significant impact on the kinetics and visco-elastic clot strength of whole blood clots formed *ex vivo*. This result is consistent with the relative low risk of bleeding observed in patients taking clopidogrel [14]. Perhaps clopidogrel has a bigger mechanistic impact on a plaque stimulated thrombus than it has on disrupting physiological hemostasis due to differences in temporal activation, sequence of thrombin activation, or the relative greater importance of platelets.

Blood component measurements indicated similar results between clopidogrel and control donors with the exception of average WBC count which was 1.78 (95% CI: 0.83 to 2.82) times higher for clopidogrel donors than for control donors. Further assessment by analysis of covariance of the difference in blood aggregation after activation with ADP and thrombin between donor groups adjusted for potential differences in WBC count resulted in two-sided p-values of p = 0.0046 and p = 0.3908 respectively leading to the same statistical conclusion as without using WBC count as a covariate (Figure 2). The similarity in remaining blood parameter measurements reduces the likelihood that a difference in another coagulation factor or blood component influenced aggregation response to adenosine diphosphate or thrombin. The averages for ACT before and after addition of heparin were similar between both donor groups. However, the addition of heparin to the blood samples did elevate the clotting time to clinically relevant values. The average clotting times for both donor groups were between 550 and 600 seconds following addition of heparin compared to averages below 200 seconds at baseline. ACTs between 300 and 600 seconds are described as the ‘safe zone’ for patients undergoing extracorporeal circulation [33] while the ACT for cardiac surgery can be targeted at 350 seconds or greater [34].

Although platelets are important for hemostasis, our results using this *ex vivo* model suggest that impaired platelet activation does not impact the effectiveness of gelatin matrices and fibrin sealants incorporating the use of thrombin. An explanation for this finding comes from one of our assays used to monitor patient compliance to therapy. A statistically significant difference at the 5% level was detected using ADP activated whole blood aggregometry which demonstrated that the clopidogrel donors had defective platelet activation compared to the control donors. This result also indicates that the donors do not harbor the polymorphism in the CYP2C19 gene that renders them poor clopidogrel responders because they do not aggregate in response to ADP [35]. However, when thrombin was used as the agonist, the average whole blood aggregometry results for clopidrogel donors were similar to that for control donors. Whole blood aggregometry is a well-established method to measure platelet function and has been found to correlate well with clinical outcome [36,37]. Therefore, these data demonstrate that thrombin can initiate platelet aggregation in platelets that are unable to respond to ADP due to clopidogrel blockade of the P2Y_12_ receptor.

The high concentration of thrombin in the thrombin based gelatin matrix and the fibrin sealant hemostats provides a possible explanation for the lack of impact of clopidogrel on their efficacy. The scientific literature supports this conclusion in that thrombin is the most potent activator of platelets and does so through a different signal transduction pathway than ADP. Thrombin activates platelets through the Par1/Par4 and GpI 7 receptors [38]. Once the signaling pathway is initiated, activation propagates though PI3 kinase and GPIIb/IIIa leading to platelet aggregation and an increase in intraplatelet calcium concentration [39]. Thus, thrombin may not only play a central role in controlling bleeding, but may also restore normal clot kinetics and strength when used in combination with gelatin matrix and fibrin sealant hemostats in patients who use prophylactic clopidogrel as a platelet aggregation inhibitor. TEG analysis of blood from donors using prophylactic clopidogrel found no detectable impact on hemostasis stimulated by either gelatin matrix or fibrin sealant hemostats which contain thrombin when compared to the control donors. This is consistent with the platelet aggregation results and clot structure analysis.

Morphological characterization of clots formed when thrombin based gelatin matrix and fibrin sealant hemostats were mixed with donor blood supports the quantitative data accumulated during this study. Although differences can be seen with FESEM based on the type of hemostat used, the fibrin morphology and incorporation of red blood cells between clots formed with clopidogrel donor blood and those of the controls were similar. The clots formed with fibrin sealant were similar to those seen previously [40,41] as well as those formed with the thrombin based gelatin matrix hemostat [42]. Therefore, not only does the use of thrombin attenuate the impact of clopidogrel by initiating platelet activation while retaining normal clot kinetics and strength, it also facilitates structurally similar clots when used in combination with gelatin matrix and fibrin sealant hemostats. However, this indicates only the potential to form a structurally adequate clot *in vivo* considering the artificial nature in which the clots were created *ex vivo*.

Whole blood was selected for testing in that it more closely reflects the conditions during surgical application of the thrombin based gelatin matrix and fibrin sealant hemostats, but there are considerations that must be addressed due to the presence of high concentration thrombin in the hemostats. While thrombin is a potent stimulator of platelets, it is also responsible for converting fibrinogen to fibrin, a reaction which also influences whole blood platelet aggregometry and TEG results. In the case of whole blood platelet aggregometry, the thrombin concentration had to be reduced from the kit concentration of 500 IU/ml to 1 IU/ml. For the purposes of simplicity it was also referred to as to platelet aggregometry even though a coagulation reaction was known to have occurred. The same can be said for the TEG assay in that the high concentration of thrombin in the hemostats elicited the same reaction making it unlikely to discern between coagulation and platelet aggregation effects by this method. However, these tests were not designed to imply a direct comparative basis for ADP stimulated platelet aggregation to thrombin stimulated platelet aggregation or to discern between coagulation and platelet aggregation effects but to evaluate the effectiveness of the hemostats when used with donor blood in which platelets were inhibited by clopidogrel.

Based on these data, thrombin based gelatin matrices and fibrin sealants are likely to be effective when used during surgical procedures involving patients currently on clopidogrel anti-platelet therapy. This is consistent with recent clinical trial data demonstrating the effectiveness of a fibrin sealant based hemostat in patients on platelet inhibitors [43]. Despite the clinically meaningful results of this study, it was limited in scope and designed to encourage additional clinical studies to verify the basic findings of this work. The small patient numbers are the main limitation of this exploratory study, reducing the power of the statistical analysis to detect differences. Therefore, analysis focused on estimation rather than on hypothesis testing. Lack of clinical correlations with this study’s TEG data is another obvious limitation. However, other research using a rabbit bleeding model to show that clopidogrel had no impact on the bleeding rate *in vivo*[44] is consistent with results in human blood presented in this paper.

This study also raises some interesting questions. In the modern era of poly-pharmacy, the impact of multiple medications such as SSRIs, SNRIs, platelet inhibitors, thrombin inhibitors, and Vitamin K antagonists as well as congenital deficiencies in hemostatic factors may have unpredictable consequences on the regulation of hemostasis. Consequently, surgical hemostats may have to be carefully evaluated in the context of this growing combinatorial complexity.

## Conclusion

The use of thrombin based gelatin matrices and fibrin sealants initiated clot formation when mixed with blood from donors on anti-platelet therapy that was similar to that of control donor blood within the constraints of the TEG assay. This study provided a basis for future investigation into the effectiveness of thrombin based hemostats during surgical procedures involving patients on anti-platelet therapy.

## Abbreviations

α: Angle; ACT: Activated clotting time; ADP: Adenosine diphosphate; CI: Confidence interval; FESEM: Field emission scanning electron microscopy; G: Shear elastic modulus strength; IFU: Instructions for use; K: Clotting time; MA: Maximum amplitude; R: Reaction time; RBC: Red blood cell; SNRI: Seritonin-norepinepherin re-uptake inhibitor; SSRI: Selective serotonin re-uptake inhibitor; TEG: Thrombelastography; TMA: Time to MA; WBC: White blood cell.

## Competing interests

The authors of this manuscript are current or former employees and consultants of Baxter Healthcare Corporation.

## Authors’ contributions

JFD was responsible for blood sample labeling and distribution, development and execution of TEG assays, organization of data for statistical analysis, drafting and submission of the manuscript, and was involved in study design. JAM was responsible for study design, maintaining the blind for the study, donor recruitment, and was involved in the drafting of the manuscript. ZY was involved in the study design, preparations of clots for FESEM, and assay execution. MH was responsible for the development and execution of the hematological assays. HR was involved in the study design and development of particle based TEG assay. MM was involved in the processing of clots for FESEM. MW was responsible for the statistical analysis. JPD was responsible for FESEM. AG was involved in study design. SD was involved in study design and drafting of the manuscript. All authors contributed to, read, and approved the final manuscript.
